# Strategies and Advancements in Harnessing the Immune System for Gastric Cancer Immunotherapy

**DOI:** 10.1155/2015/308574

**Published:** 2015-10-22

**Authors:** Vinod Vijay Subhash, Mei Shi Yeo, Woei Loon Tan, Wei Peng Yong

**Affiliations:** ^1^Cancer Science Institute of Singapore, Yong Loo Lin School of Medicine, National University of Singapore, Singapore 119228; ^2^Department of Haematology-Oncology, National University Hospital, Singapore 119228

## Abstract

In cancer biology, cells and molecules that form the fundamental components of the tumor microenvironment play a major role in tumor initiation, and progression as well as responses to therapy. Therapeutic approaches that would enable and harness the immune system to target tumor cells mark the future of anticancer therapy as it could induce an immunological memory specific to the tumor type and further enhance tumor regression and relapse-free survival in cancer patients. Gastric cancer is one of the leading causes of cancer-related mortalities that has a modest survival benefit from existing treatment options. The advent of immunotherapy presents us with new approaches in gastric cancer treatment where adaptive cell therapies, cancer vaccines, and antibody therapies have all been used with promising outcomes. In this paper, we review the current advances and prospects in the gastric cancer immunotherapy. Special focus is laid on new strategies and clinical trials that attempt to enhance the efficacy of various immunotherapeutic modalities in gastric cancer.

## 1. Introduction

Gastric cancer is the second leading cause of cancer-related deaths worldwide and is among the most frequent malignant tumors in East Asian countries [1]. The disease is generally asymptomatic and is diagnosed often at late stage, resulting in metastasis of cancer that can progress to an advanced and even terminal stage. For early-stage gastric cancer, surgical resection remains the mainstay of curative-intend treatment [2]. Treatment is largely palliative for advanced disease and consists of chemotherapy and radiation. Despite decades of research in newer systemic therapies, the combination of a fluorinated pyrimidine with a platinum agent remains the effective chemotherapy standard [3]. Although use of oral fluorinated pyrimidines (e.g., oxaliplatin) has improved therapy convenience and lessened toxicity, the overall survival in advanced gastric cancer has not been significantly improved over the past few decades. The second line treatment using taxanes and irinotecan also shows modest survival benefits and treatment tolerance [4].

The recent developments in targeted molecular therapies including selective targeting of human epidermal growth factor receptor 2 (HER2) and vascular endothelial growth factor (VEGF) have shown significant advances in gastric cancer treatment. The TOGA trial using anti-HER2 antibody trastuzumab met not only the primary endpoint of improved overall survival but also the secondary endpoint of improved response rates and progression-free survival [5]. However, the benefit of this approach is limited to patients with HER2-positive or HER2-amplified tumors [6]. The REGARD and RAINBOW trials using VEGF targeting antibody ramucirumab have also shown significant increase in the overall survival of patients with advanced-stage gastric and gastroesophageal junction adenocarcinoma [7, 8]. Still, therapeutic options in gastric cancer remain very limited as other candidate therapies targeting epidermal growth factor receptor [9, 10], platelet-derived growth factor receptor [11], c-Met (NCT01697072), and fibroblast growth factor receptor 2 (NCT01457846) have shown little success in advanced disease. Recent knowledge regarding the immune regulatory mechanisms and tumor microenvironment presents us with novel strategies in anticancer therapeutics. One of the most recent and promising approaches is “immunotherapy” with documented clinical responses in diverse tumor types. The field of immunotherapy focuses on developing therapeutic strategies that would enable the immune system to achieve durable and adaptable cancer control. Recent studies have shown the significance of specific immune suppressive mechanisms that would act as either part of the tumor or the immune system to suppress antitumor responses. The astonishing outcomes of immunotherapy in melanoma have kindled great interest in reviving similar strategies in other cancers, including gastric cancer [12]. The scope of this review is to discuss strategies adopted in gastric cancer immunotherapy and to provide an overview about its recent advances and future prospects.

## 2. Immune Surveillance and Evasion of Immune Response in Cancer

**Figure 1:**
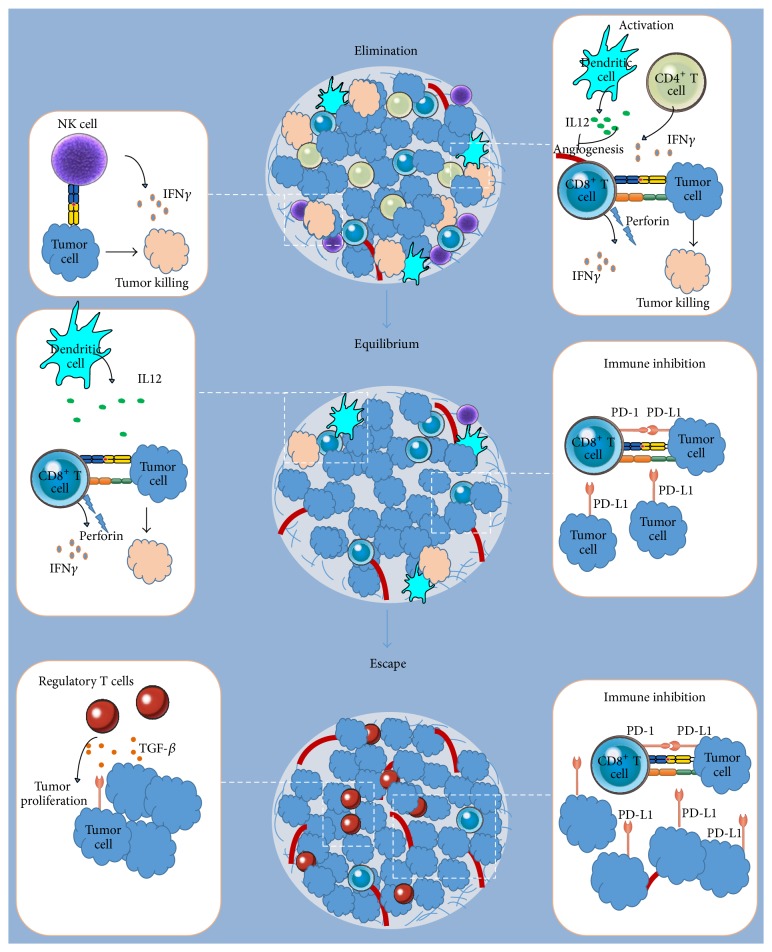
Immunosurveillance in cancer. Elimination phase: immune effector cells are recruited and tumors are destroyed; equilibrium phase: cells that survive elimination undergo chronic maintenance and genetic adaptation in an immunosuppressive environment; escape phase: the tumor microenvironment becomes immunosuppressive and cancer becomes poorly immunogenic.

The ability of the immune system to detect tumor cells as nonself and eliminate them before developing into a clinical malignancy is called “immunosurveillance” [13]. However, tumor cells are armed with several mechanisms that help them to modulate the immune system and avoid detection by immune effector cells. Downregulation of HLA proteins (classes I and II) and molecules that facilitate antigen processing and presentation is a common characteristic in tumors [14]. Furthermore, tumor cells may express immune checkpoint ligands, such as PD-L1 either through constitutive oncogene-driven expression or through upregulation in response to interferon- (IFN-) *γ* released by T cells at the tumor site [15]. Immune surveillance functions through a mechanism of “immunoediting” and has an integral and complex role in cancer biology. Immunoediting plays a dual role in cancer by promoting tumor growth and mediating the eradication of disease. Understanding this seemingly contradictory role requires a deeper insight into the dynamic interplay between various immune effector cells, tumor cells, stromal cells, and soluble factors [16]. In cancer biology, the entire process of immunoediting goes through three key phases: elimination, equilibrium, and escape [17] (Figure 1). In the elimination phase, growing tumors are detected by the innate and adaptive immune cells (natural killer cells, CD8^+^ and CD4^+^ T cells) that recognize remodeling of stroma and changes in the microenvironment. These immune cells secrete interferon gamma and cytokines which inhibit angiogenesis and tumor cell proliferation. Dendritic cells (DCs) are also recruited to the tumor site that secretes cytokines and present processed tumor antigens to T cells. Eventually, the CD4^+^ and CD8^+^ T cells identify the antigen-bearing tumor cells and destroy them. The tumors that survive the elimination phase enter the longest of the three phases, equilibrium phase. In this phase, the DCs, tumor cells, and CD8^+^ T cells remain in a state of dynamic balance and the tumor cells remain quiescent under pressure from the immune system. During this process, the highly heterogenic and genetically unstable tumors may survive and acquire resistance, and this leads to the escape phase. In the escape phase, the regulator T cell (T reg) mediates immunosuppression and immune effector cells undergo apoptosis. The tumor immunogenicity could also be shut by high levels of immune suppressive cytokines including transforming growth factor-*β* (TGF*β*), tumor necrosis factor-*α* (TNF*α*), and IL10, in the tumor microenvironment [18].

## 3. Tumor Microenvironment and Immunogenicity in Gastric Cancer

A microenvironment rich in inflammatory cells is an essential component of epithelial tumors and malignancies that develop in an inflammation dependent or independent manner. The tumor microenvironment is composed of multiple components such as tumor parenchymal cells, lymphocytes, fibroblast, mesenchymal cells, angiogenic factors, and more [22]. Cancer cells secrete various growth factors and proteases that together activate and modify their microenvironment. On the other hand, stromal cells also exert their effect on cancer cells by secreting soluble factors such as growth factors and cytokines [23]. The whole microenvironment is dynamic in nature in which each component is capable of interacting extensively with each other, thereby contributing to tumor development and progression [24]. Cell-mediated immunity to tumor development is based on an effective interaction between macrophages and T cells [25]. The components of the adaptive immune system play a major role in anticancer immunity. In gastric cancer patients, intratumoral infiltration of cytotoxic T cells and memory T cells was shown to be associated with better prognosis and survival [26]. In the gastric tumor microenvironment, macrophages constitute one of the most abundant immune populations and those recruited to the tumor stroma are known as the tumor associated macrophages (TAMs). TAMs infiltration leads to immunological inactivity of T cells in gastric cancer and is an indication of poor prognosis [27, 28]. Although the biology and immunogenicity of gastric cancer largely remain uncharacterized, recent strategies to harness the immune system for antitumor efficacy seem to offer the potentiality for effective clinical management and possibly cure of this malignancy.

## 4. Immunotherapy in Gastric Cancer

In cancer immunotherapy, an effective immune-based strategy requires the induction of effective tumor-specific immunity. Such a strategy should eliminate immunological tolerance to the tumor and induce antitumor immunity [29]. An effective antitumor response is mounted when specialized cytotoxic cells are induced to recognize and directly attack tumor cells based on expression of antigens on the tumor cell surface. Based on these concepts, several studies are underway in preclinical and in clinical trials that explore the therapeutic utility of immune harnessing in gastric cancer. Here we discuss the current approaches, advances, and clinical trials which are underway in gastric cancer immunotherapy.

## 5. Innate and Adaptive Immune Cells in Gastric Cancer Immunotherapy

### 5.1. Dendritic Cell (DC) Based Vaccines

Cancer vaccines are designed to further enhance the ability of immune system to seek and destroy tumors by activating tumor-specific T cells. Antigen-presenting cells (APCs) induce the T cell activation by presenting peptides derived from tumor associated antigens to T cells. DCs are the most powerful APCs at the interphase between innate immunity and adaptive immunity with the ability to activate many other effector cells including NK, B, and NKT cells [29, 30]. The antigens processed by DCs are loaded onto the MHC class I molecule for presentation to CD8^+^ cytotoxic T cells or to MHC class II molecules for presentation to CD4^+^ helper T cells. These characteristic features generate great interest in using DCs as ideal candidates for cancer immunotherapy [31]. Ishigami et al. [32] demonstrated lower lymph node metastases and lymphatic invasion in patients with high DCs infiltration as compared to patients with low DCs infiltration. Moreover, the 5-year survival rate was significantly higher (78% increase) in patients with high DCs infiltration as compared to patients with less DCs infiltration. These reports were suggestive of the therapeutic utility of DC based tumor vaccines as an effective form of immune-adjuvant therapy in gastric cancer. Of the 325 trials reported in https://clinicaltrials.gov/ on DC therapy [33], six studies involve GC patients (Table 1), with only two having published results. Recent studies have investigated the implications of tumor associated antigens as targets for vaccine development. The tumor associated antigens act as immune-regulators by reducing the infiltration of T reg cells and other immune-mediators (e.g., TGF-*β*) [34, 35]. Therapeutic vaccines using HER-2/neuropeptide pulsed DCs have shown significant tumor regression in gastric cancer patients [36]. Similarly, vaccines using MAGE-A3 peptides pulsed DCs have also shown promising results with induction of peptide specific T cell response and minor tumor regression in some patients [37]. However, clinical applications of current DC vaccines have been limited due to their short lifespan. The activated CD8^+^ cytotoxic T lymphocytes acquire cytolytic activities leading to the early removal of DCs and thereby limit the ability of DCs to prime and expand CTL immunity [38, 39]. Studies have suggested ways to enhance the efficacy of DC based antitumor vaccines. For example, DCs modified with granulocyte-macrophage colony-stimulating factor (GM-CSF) gene tend to be more matured with enhanced capacity to activate proliferation of T lymphocytes [40]. Recently, Kim et al. [41] demonstrated that the efficacy of DC vaccines can be enhanced by siRNA mediated targeting of PTEN, a negative regulator of PI3K/AKT pathway. PTEN downregulation resulted in AKT dependent maturation of DCs that in turn lead to enhanced expression of costimulatory molecules. Immunosuppressive factors, such as IL-10, secreted by DCs (or other regulatory cells) downregulate the DCs functionality by inhibiting specific surface receptors (e.g., IL-10R). Recent data showed that the targeting IL-10 receptor with siRNA can increase the effectiveness of DC based vaccine [42].

### 5.2. Vaccines from Tumor Associated Antigens

In gastric cancer, targeting the gastrin peptide has been studied in a multicenter, phase II trial [43]. Patients with gastroesophageal adenocarcinoma or untreated metastatic unresectable gastric cancer received G17DT (Aphton) vaccination containing 9-amino-acid epitope derived from the aminoterminal sequence of gastrin-17 and cisplatin plus 5-fluorouracil. 61% of the total 94 patients treated were deemed to be immune responders based on two consecutive anti-gastrin antibody titers of at least 1 unit. These patients had a longer time to progression and a longer median survival rate compared to nonresponders [44]. Another trial in gastric cancer patients involved* in vitro* testing of peripheral blood mononuclear cells (PBMCs) against peptides on HLA-A24 and HLA-A2 type. Patients vaccinated with these peptides have shown successful induction of HLA-A24 restricted or HLA-A2 restricted and tumor-specific CTL activity in PBMCs. Here, 50% of the vaccinated patients had increased cellular and humoral immune responses to the vaccinated peptides in postvaccination PBMCs [45]. Peptides targeting other tumor associated antigens have also been used to stimulate specific immune response in gastric cancer. MAGE-3 peptide/chitosan-deoxycholic acid vaccine-loaded nanoparticles have been shown to simulate an antitumor immune response and tumor regression in mouse models of gastric cancer [46]. A recent study has shown combination therapy using cancer vaccines and standard chemotherapy as a promising strategy for the treatment of advanced gastric cancer. Peptides derived from human vascular endothelial growth factor (VEGF) receptor 1 and vascular endothelial growth factor receptor 2 combined with chemotherapy (S-1 plus cisplatin) have been shown to induce a VEGF specific cytotoxic lymphocyte response in patients with advanced gastric cancer resulting in a partial response in 55% of patients as well as prolonged overall survival [47].

### 5.3. Therapies Using Natural Killer (NK) Cells

NK cells prevent the metastatic dissemination of human cancer and thus play a major role in antitumor response [48]. This presents us with different modalities to combat the malignancy by manipulating the NK cells “arm,” but it is hampered by limited knowledge on NK cell subpopulations and functionality, exiguous amounts of blood NK cells, and difficulties associated with its large scale production [29, 33]. The primary objective in current NK cell based immunotherapy is to overcome the NK cell paralysis. Several approaches are being tested, where one approach involves the use of* in vitro* expanded allogeneic NK cells for adoptive immunotherapy. Contrary to autologous NK cells, allogeneic NK cells have the advantage of not being inhibited by self-histocompatibility antigens. The utility of stable allogeneic NK cell lines is also being investigated for adoptive transfer therapy, owing to its feasibility for quality controls and large scale production. Another approach targets genomic manipulation of NK cells or NK cell lines to enhance the expression of Fc receptors and/or chimeric tumor-antigen receptors and cytokines [49]. For gastric cancer immunotherapy, NK cells can be expended from PBMCs of healthy individuals (in the presence of K562 cells expressing membrane bound IL-15 and 4-1BB Ligand) and from patients with different solid tumors [50]. Adoptive transfer of NK cells presents a promising avenue for immunotherapy as studies have shown a strong correlation between high levels of tumor infiltrating NK cells (TINKs) and favorable tumor outcome in patients with gastric carcinoma, colorectal carcinoma, and squamous cell lung cancer. This suggests the NK cell infiltration as a positive prognostic marker in cancer [32, 51, 52]. Rosso et al. confirmed the association of NK cells with survival in gastric adenocarcinoma as high concentration of NK cells correlated with better patient survival, especially in advanced stage [53]. More interestingly, Saito et al. demonstrated high frequency of apoptotic NK cells in gastric cancer patients that correlated with cancer progression as compared to normal controls [54]. Recent studies have evaluated the curative action of lupeol, triterpene against many diseases. Lupeol was shown to favor the proliferation and cytolytic ability of NK cells against gastric cancer cells and hence could be used in therapies in combination with adoptive transfer of NK cells [55].

### 5.4. T Cell Based Adoptive Transfer Therapy

In adoptive T cell therapy, tumor-specific T cells are isolated from a patient and amplified* in vitro*. This allows manipulation of the T cells by priming the cells to tumor antigens or genetic modification tumor antigens [56]. The primed cells are subsequently reinfused into the patient in large numbers. Despite having no FDA approved protocols yet, the hopes on and prospects of adoptive T cell therapy are at a high level. With a deeper insight into the cancer biology and better understanding of T cell functionalities, adoptive T cell therapy could mark significant advances in the treatment of gastric cancer patients. Although the adoptive transfer using lymphodepleted hosts [57] and the immunosuppressive Tregs [58] have shown promising outcomes, the therapeutic utility of these strategies still has to be validated clinically. The major types of T cell based anticancer therapeutic protocols, using (a) cytotoxic T lymphocytes, (b) tumor-infiltrating lymphocytes, and (c) chimeric antigen receptor- (CAR-) expressing T cells are further described here.

#### 5.4.1. Cytotoxic T Lymphocytes (CTLs)

Improved CTL cell culture technology [59] has aided the first clinical tests for adoptive transfer of CTLs in melanoma patients that resulted in substantial antitumor immune response. This was further confirmed in another trial by Mackensen et al. in which T cell transfer in patients resulted in CTL's engraftment that was detectable for a period of two continuous weeks [60]. Studies by Kim et al. showed* ex vivo* expansion of tumorolytic T cells in gastric cancer [61]. In this approach, human peripheral blood mononuclear cells were cultured in medium with IL-2 and anti-CD3 antibody. The resulting heterogeneous population were called cytokine induced killer (CIK) cells which were mostly CD3^+^ T cells (97%) comprising 1% CD3 CD56^+^, 36% CD3^+^ CD56^+^, 11% CD4^+^, and 80% CD8^+^. The CIK cells were able to induce high amounts of IFN-*γ* and moderate TNF-*α* and caused significant growth inhibition of MKN74 gastric cancer cells [62, 63]. Collectively, these studies add greater credence to the potentiality of CTL therapy in gastric cancer. In advanced gastric cancer, combinational therapies using CIK cells and chemotherapeutic drugs have shown greater benefits. In fact, the patients cured with the combinational therapy showed a significant decrease of serum levels of the cancer markers and a marked improvement of life quality, in comparison to patients treated with chemotherapy alone [64, 65]. An effective and minimally invasive approach of adoptive cellular therapy was showed by Du et al. in mouse models of gastric cancer. Here, peritumoral injection of CIKs resulted in considerable tumor infiltration with very small CIK intratumoral accumulation [66].

#### 5.4.2. Tumor Infiltrating Lymphocytes (TILs)

TILs based adoptive transfer therapy requires T cell isolation from surgical tissues or neoplastic biopsies. This is followed by* ex vivo* selection of tumor-specific T cells. The technique requires about 6 weeks before the T cells would be ready for infusion [67]. However, only less than 40% of the biopsies yield satisfactory T cell population. This poses additional barriers for TIL based randomized clinical trials. The study showed promising results in preclinical models [68]; however, the results from clinical experiments were less encouraging [36, 69] except for in melanoma patients. Of interest is a clinical study of adoptive immunotherapy with TILs in combination with chemotherapy in gastric cancer that resulted in longer than 50% survival than chemotherapy alone [36]. Recent data indicate that the presence of TILs positively correlates with patient survival in ovarian and colorectal cancer [70, 71] and have an important role in pancreatic cancer [72]. This prompts further enforcement of this protocol for other usually encountered epithelial cancers, provided technical limitations of current tissue culture approaches are overcome. A recent study that analyzed the T cell response to gastric cancer associated antigenic peptides has laid the ground work for possible vaccination of gastric cancer patients with tumor associated antigenic peptide [73]. But, in order to get tumor cell killing* in vivo*, the activity of TH1 cells specific to cancer peptides needs to be enhanced by injection of tumor peptide-specific T cells expanded* in vitro* [29]. Another strategy in cancer immunotherapy is genetic modification of T cells to improve antitumor effects. Although there is little clinical experience with engineered T cells for cancer therapy, it is notable that clinical trials to date using cells engineered to express suicide molecules indicated that the approach is safe [29, 59]. Another purpose of engineered T cells is to enhance survival of CTLs, because they have short-term persistence in the host without antigen-specific T helper cells and/or cytokine infusions [74]. One of the primary limitations in adoptive T cell therapy in some tumors is their poor antigenicity; therefore, neither T cells with high avidity for tumor-specific antigens nor T cells with the desired specificity remain in the patient following chemotherapy. Studies have suggested strategies to overcome this limitation by endowing T cells with chimeric receptors that have antibody based external receptor structures and cytosolic domains that encode signal transduction modules of the T cell receptor [75].

#### 5.4.3. Chimeric Antigen Receptor- (CAR-) Expressing T Cells

The adoptive transfer of chimeric antigen receptor- (CAR-) expressing T cells is another example of cancer immunotherapy that is relatively new and promising. This form of therapy is based on the genetic engineering of T cells through the introduction of a chimeric antigen receptor (CAR), which redirects T cells to specific tumor associated antigens (TAA) on malignant cells [76]. The effectiveness and safety of CAR-expressing T cells are primarily determined by the choice of target antigen [77]. A tumor associated antigen which is important for cell survival will have little chance for immune editing and tumor escape and hence would serve as an ideal target to obtain maximum therapeutic effect [78]. Tumor targeting using CAR- T cells is not restricted to HLA molecules and therefore is applicable to a broader range of patients regardless of their HLA type [17]. CARs also facilitate genetically modified T cells to react to a wider range of molecules as they are capable of recognizing any cell surface antigen, ranging from proteins and carbohydrates to glycolipids [79]. Furthermore, unlike tumor-infiltrating lymphocytes which are difficult to produce* in vitro* in sufficient number within a short period of time, tumor-specific T cells are easier to produce in large amount within a moderately short period of time, thus making it a more attractive technique to be used under clinical settings [80]. Genetically engineered T cells have already been successfully developed against a wide range of tumor antigens including CD19 [81], ERRB2 [82], CAIX [83], and MUC16 [84]. A phase I clinical trial is currently underway to verify the safety of CEA targeted CAR T cells for the treatment of CEA positive gastric, lung, colorectal, and breast cancers (NCT02349724).

## 6. Checkpoint Inhibition in Gastric Cancer Therapy

**Figure 2:**
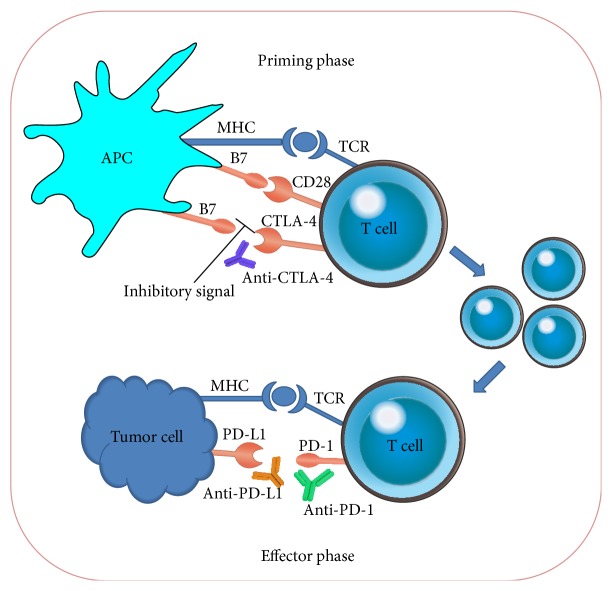
Immune checkpoint targeting. T cells recognize MHC-antigen complex through the primed T cell receptor (TCR) and CTLA-4 competes with CD28 to bind to costimulatory molecule B7 on antigen-presenting cells (APCs). This causes the initiation of an inhibitory signal that leads to suppression of T cell activation resulting in the failure to induce an effective antitumor responses [19]. Anti-CTLA-4 antibodies block this inhibitory signal and thus enhance the antitumor immunity through T cell proliferation and tumor-specific cytotoxicity. During the effector phase of T cell response, programmed death-1 (PD-1) inhibitory receptor is expressed by T cells after antigen exposure and its ligation with PD-L1 ligands expressed on tumor cells results in negative regulation of T cells [20]. Blocking of antibody mediated blockade of PD-1 or PD-L1 has been shown to enhance T cell activity with specificity for tumor cells [21].

A promising avenue of immunotherapeutic research in cancer is the use of immune checkpoint inhibitors that targets specific molecules serving as checks and balance in the regulation of immune response [85]. Immune checkpoints are the coinhibitory molecules, essential for the maintenance of self-tolerance to prevent immune overactivation and host tissue damage under normal physiologic conditions [86]. As the name implies, inhibitory molecules mediate negative signals that modify MHC-TCR (major histocompatibility complex-T cell receptor) signalling pathways and thus regulate T cell survival, proliferation, differentiation, or responsiveness to cognate antigens [87]. Employing this inhibitory pathway, the immune system can attenuate excessive immune reactions and ensure self-tolerance, which is important for maintaining immune homeostasis [88]. During malignant transformation, cancer takes advantage of this ability to evade the immune system by inhibiting T cells, which are specific for tumor antigens [89]. Blockade of the immune checkpoints by antibodies or modulation by recombinant forms of ligands or receptors (commonly called checkpoint inhibitors) can significantly enhance anticancer immunity or reawaken silenced immune responses [15]. Therapies using checkpoint inhibitors promises a systemic approach to achieve durable response or even cure in gastric cancer and other malignancies. While a variety of agents could be deemed to interact with immune checkpoint inhibitors, the focus of this review is limited to the most advanced agents in clinical trials for gastric cancer immunotherapy. Checkpoint inhibitors that augment the anticancer immune response in gastric cancer include T lymphocyte antigen- (CTLA-) 4, anti-programmed death- (PD-) 1, and anti-PD ligand 1 (PD-L1) (Table 2).  Figure 2 provides a schematic representation of the action of immune checkpoint inhibitors in cancer immunotherapy [88].  Table 3 summarizes the representative checkpoint inhibitors that were clinically tested/being tested in gastric cancer patients.

### 6.1. CTLA-4

CTLA-4 is a member of the CD28 immunoglobulin superfamily (IGSF) of receptors [90]. CTLA-4 is expressed on the surfaces of activated conventional CD4^+^ and CD8^+^ T effector cells and on CD25^+^FOXP3^+^ T regulatory cells [19, 88]. In fact, higher levels of surface expression of CTLA-4 are seen as T reg cells as compared with effector T cells [91]. CTLA-4 binds to ligands B7.1 (CD80) and B7.2 (CD86) on APCs, where it competes with costimulatory receptor CD28 to bind with shared ligand [88]. As CTLA-4 binds with higher affinity than CD28, it reduces CD28-dependent costimulation. CTLA-4 also mediates direct inhibitory effects on the MHC-TCR pathway and suppresses antitumor immune activities in the tumor microenvironment [92]. Under normal circumstances, CTLA-4 expressed on the surface of T-lymphocytes transduces an inhibitory signal upon antigenic recognition by TCR. An anti-CTLA-4 antibody would block such an inhibitory signal, thereby enhancing the interaction of CD28 with its ligand B7 on the tumor cell [91]. Observations made to date suggest that anti-CTLA-4 antibodies function not only by blocking inhibitory signals from reaching effector T cells but also by depleting the T reg cells present in the tumor microenvironment [93]. Preclinical studies in animal models using anti-CTLA-4 antibody showed significant antitumor responses without serious immune toxicities [94–96]. However, CTLA-4 inhibition in mice bearing low-immunogenic tumor did not show any significant tumor regression. Interestingly, combination of CTLA-4 blockade with a cellular vaccine transduced with granulocyte-macrophage colony-stimulating factor (GM-CSF) leads to significant tumor regression in low-immunogenic tumors [95]. Hence, tumors with an endogenous anti-tumor immune response appear to be a better candidate for CTLA-4 blockade therapy [88]. Based on these preclinical studies, two anti-CTLA-4 antibodies, ipilimumab and tremelimumab, have been developed for use in humans.

Tremelimumab is a fully human IgG2 monoclonal antibody that blocks the binding of B7-1 and B7-2 to CTLA-4 resulting in the inhibition of B7-CTLA-4 mediated downregulation of T cell activation and enhances anticancer immunity. The anticancer efficacy of tremelimumab was tested in a small phase 2 trial, with tremelimumab as a second-line therapy in 18 patients with gastric cancer [97]. Although the objective response rate was 5%, the median survival was 4.8 months and, therefore, similar to that expected with other chemotherapies in gastric cancer. Despite having a low objective response rate of 5%, the median overall survival at 4.8 months is similar to that expected with other chemotherapies in gastric cancer, in which the average reported median survival was 5.6 months (range 2.5–11 months [98]). At 12.2 months, survival for patients who had stable disease following tremelimumab compares favorably with that of chemotherapy responders whose mean survival was 9.1 months (range 5.5–12 months). However, patients with enhanced proliferative response to carcinoembryonic antigen (CEA) showed a higher median survival (17.1 months) as compared to patients who are nonresponsive to CEA (4.7 months). Hence, combining of CTLA-4 blockade with antigen targeting could too be an alternative strategy that would enhance the efficacy of checkpoint inhibition therapies. In advanced gastric cancer, first line systemic CTX therapy is considered standard of care (SOC). However, CTX therapy generally encompasses severe adverse effects (SEAs) and disease relapse. A recent phase 2 clinical trial investigated the supporting role of CTLA-4 based immunotherapy in advanced gastric cancer (NCT01585987). This study compared the efficacy of ipilimumab with the existing SOC therapy and explored a new maintenance concept by using sequential administration of ipilimumab in patients with unresectable locally advanced or metastatic gastric or gastroesophageal junction (GEJ) cancer. The study was recently completed and results are yet to be availed.

### 6.2. PD-1 and PD-L1

PD-1 is another coinhibitory receptor expressed on the surface of activated T cells, T reg cells, and monocytes. PD-1 induces a negative regulation of effector T cells by interacting with its ligands PD-L1 and PD-L2 on the tumor cells. The predominant ligand, PD-L1, is expressed on many tumors and suppressive immune cells in the tumor microenvironment and participates in tumor immune evasion. Interaction of PD-1 and PD-L1 results in the inhibition of T cell functioning [99]. As a result, T cells have a decreased ability to produce cytokines, proliferate, or cause tumor lysis. Antibody mediated blockage of PD-1 or PD-L1 results in the inhibition of this checkpoint, leading to T cell activation and enhanced antitumor activity. Recent studies provide key experimental evidence on the emerging and critical role of immunotherapeutic antibodies that can block PD-1/PD-L1 interactions, in the treatment of cancer [100, 101]. The anti-PD-1 and PD-L1 antibodies have generated much interest in gastric cancer immunotherapy since the first data for this class of agents in gastric cancer were presented at ESMO 2014.

Pembrolizumab is an anti-PD-1 antibody that to date has shown good antitumor activity and good safety and tolerability in multiple tumor types. Data from the gastric cohort of the Phase Ib (KEYNOTE-012) trial evaluating pembrolizumab in PD-L1-positive advanced solid tumors were presented at ESMO 2014. In this study, 40 percent of patients were defined as PD-L1-positive by IHC staining. Efficacy data presented showed that overall response rate (ORR) was 30.8 percent and disease control rate (DCR) 43.6 percent [102]. Study by Herbst et al. [100] evaluated the single-agent safety, activity, and associated biomarkers of PD-L1 inhibition using the MPDL3280A, a humanized monoclonal anti-PD-L1 antibody in patients with locally advanced or metastatic solid tumors or hematological malignancies [103]. Across multiple cancer types, responses as per RECIST v1.1 (Response Evaluation Criteria in Solid Tumors, version 1.1) were observed in patients with tumors expressing relatively high levels of PD-L1. Although varied, confirmed objective responses were observed in many cancers including gastric cancer. Another antibody that blocks the interaction between PD-1 and the corresponding ligand PD-L1 is nivolumab. Nivolumab has shown encouraging efficacy in many tumor types. In December 2014, the U.S. Food and Drug Administration (FDA) approved the use of nivolumab for the treatment of patients with unresectable or metastatic melanoma. More recently, nivolumab has been licensed as a second-line therapy in squamous non-small cell lung cancer based on an improvement in overall survival compared with docetaxel (9.2 months versus 6.0 months, HR 0.59; *P* = 0.00025 [NCT01642004]). A phase I trial using PD-L1 inhibitor MED14736 in combination with tremelimumab is currently undergoing in gastric cancer and in other solid tumors (NCT01975831).

Preclinical data showed that the dual blockade of PD-1 and CTLA-4 was associated with increased cytokine release and increased proliferation of CD8^+^ and CD4^+^ T cells when compared with single receptor blockade [104, 105]. An ongoing phase Ib/II trial is investigating the activity of single-agent nivolumab and nivolumab plus ipilimumab in patients with metastatic gastric cancer, pancreatic cancer, triple-negative breast cancer, and small cell lung cancer (NCT01975831). A phase 1b/2 study of MEDI4736, a human immunoglobulin (Ig) G1к anti-PD-L1 antibody as monotherapy or in combination with CTLA-4 inhibitor tremelimumab monotherapy in gastric or GEJ adenocarcinoma is also currently underway (NCT02340975). Preclinical data showed that the dual blockade of PD-1 and CTLA-4 was associated with increased cytokine release and increased proliferation of CD8^+^ and CD4^+^ T cells when compared with single receptor blockade [104, 105]. An ongoing phase Ib/II trial is investigating the activity of single-agent nivolumab and nivolumab plus ipilimumab in patients with metastatic gastric cancer, pancreatic cancer, triple-negative breast cancer, and small cell lung cancer (NCT01975831).

## 7. Future Prospects

Tumor heterogeneity is often observed in cancer due to the accumulation of multiple gene mutations. Furthermore, the host immune cell repertoires in a tumorigenic environment are also diverse and heterogeneous. These phenomenons have made it significantly challenging to design effective treatment strategies for cancer patients [106]. The specificity, adaptability, and memory response that are inherent to the immune system give us the opportunity to explore immunotherapeutic strategies to measure multiple components, not just a single biomarker, that can be targeted overtime to provide curative treatments to cancer patients [107]. In the coming years, with longer follow-up periods, the completion of ongoing trials, and development of new targeted agents, the landscape of immunotherapy is likely to get richer but more complicated [108, 109]. However, careful evaluation of immune responses to tumors and normal tissue during the application of immunogenic agents is necessary to achieve the desired anticancer immunity while maintaining immunologic tolerance to self-antigens expressed on normal tissue cells to avoid autoimmune response.

Rapid advances in understanding of the details of the molecular events and regulatory pathways involved in effective use of cytotoxic cells as antitumor therapy have prompted work on developing customized or engineered cells. Thus, the future of immunotherapy also lies in developing genetic tools that could engineer and enhance T cell specificity and function. Although chimeric antigen receptors offer a promising new therapeutic method, selection of candidate target antigens is essential for improved efficacy and safety of the chimeric antigen receptor-based therapy [13]. The Cancer Genome Atlas Research Network recently analyzed the molecular characteristics of gastric adenocarcinoma, identifying four tumor subtypes. The Epstein-Barr virus subgroup showed elevated PD-L1 expression, suggesting the robust presence of immune cells and supporting the use of immune checkpoint inhibitors in gastric cancer [110]. Recent clinical trials have actively investigated the potential for synergistic effects by combining immune checkpoint inhibitors with other agents. The partner agents/therapies include other checkpoint agents, cytotoxic agents, anticancer vaccines, cytokines, and radiotherapy [88]. Epigenetic alterations also play a pivotal role in cancer development and progression. Novel drugs that possess significant immunomodulatory properties are in development and could induce reversion of epigenetic alterations. Use of this knowledge, together with the availability of new and highly effective immunotherapeutic agents, allows us to plan for highly innovative proof-of-principle combination studies that will likely open the path to more effective anticancer therapies [111]. A recent study evaluated the correlation of benefit with DNA mismatch repair (MMR) deficiency as a genetic guide for immunotherapy treatment in colorectal cancer (KEYNOTE-164). The data presented at ASCO 2015 Annual Meeting showed promising results and open doors in utilizing MMR as a clinical tool for patient stratification in immunocheckpoint therapies. Oncolytic viruses that are designed to selectively replicate in and lyse tumor cells offer to enhance systemic and regional antitumor immunity and thus emerges as a promising approach in cancer patients. Recently, a survivin promoter-regulated oncolytic adenovirus with Hsp70 gene was shown to exert effective antitumor efficacy in gastric cancer immunotherapy [112]. A phase 1 study of CEA specific CTLs induced by dendritic cells infected by recombinant adeno-associated virus with CEA gene in the treatment of late stage gastric cancer is currently underway (NCT02496273). Next generation antibodies targeting 4-1BB/CD127 receptors is also being tested as potential therapeutic agents in various solid tumors (NCT01307267).

## 8. Conclusion

Immunotherapy in gastric cancer is still evolving and is limited by the lack of gastric-specific biological exploration. To enhance the robustness of gastric cancer immunotherapeutic approaches, future studies should further characterise the intrinsic immune escape mechanisms, adopted specifically by the gastric cancer cells. Advances in immunotherapy and biomarker research will likely drive the future of gastric cancer as this indication begins to move away from chemotherapy and towards targeted and personalised therapy, thanks to an improved molecular understanding of this complex disease. With a deeper insight of the immune biology and tumor microenvironment and better design of targeted and combinational approaches, future of immunotherapy surely envisages the best possible outcome in gastric cancer treatment.

## Figures and Tables

**Table 1:** Dendritic cell vaccines in gastric cancer immunotherapy.

Reference	Title	Status
NCT00004604	A phase I study of active immunotherapy with carcinoembryonic antigen (CEA) RNA-pulsed, autologous human cultured dendritic cells in patients with metastatic malignancies expressing carcinoembryonic antigen	Completed

NCT00005956	A pilot study of active immunotherapy with HER2/neu intracellular domain protein pulsed, autologous, cultured dendritic cells in patients with no evidence of disease after standard treatment for HER2/neu expressing malignancies	Completed

NCT00027534	A phase I study of active immunotherapy with autologous dendritic cells infected with CEA-6D expressing fowlpox-tricom in patients with advanced or metastatic malignancies expressing CEA	Completed

NCT01522820	A phase I clinical trial of mTOR inhibition with rapamycin for enhancing intranodal dendritic cell vaccine induced antitumor immunity in patients with NY-ESO-1 expressing solid tumors	Ongoing

Kono et al. [36]	Dendritic cells pulsed with HER-2/neu-derived peptides can induce specific T cell responses in patients with gastric cancer	Completed

Sadanaga et al. [37]	Dendritic cell vaccination with MAGE peptide is a novel therapeutic approach for gastrointestinal carcinomas	Completed

**Table 2:** Checkpoint inhibitors in gastric cancer immunotherapy.

Target	Agent	Description
CTLA-4	Ipilimumab	Humanized IgG1 kappa
	Tremelimumab	Fully human IgG2

PD-1	Nivolumab	Fully human IgG4
	Pembrolizumab	Humanized IgG4

PD-L1	MPDL3280A	Human IgG1
	MEDI4736	Human IgG1 kappa

**Table 3:** List of clinical trials in gastric cancer using immune checkpoint inhibitors.

Reference	Title	Sponsor and collaborations	Inhibitor target	Drug	Phase	Status	Duration
NCT02494583	Randomized, Active-Controlled, Partially Blinded, Biomarker Select, Phase III Clinical Trial of Pembrolizumab as Monotherapy and in Combination With Cisplatin + 5-Fluorouracil Versus Placebo + Cisplatin + 5-Fluorouracil as First-Line Treatment in Subjects With Advanced Gastric Gastroesophageal Junction (GEJ)	Merck Sharp & Dohme Corp.	PD-1	Pembrolizumab	3	Recruiting	Study first received: June 8, 2015 Estimated completion date: April, 2019

NCT02335411	A Phase II Clinical Trial of Pembrolizumab as Monotherapy and in combination with Cisplatin + 5-Fluorouracil in Subjects with Recurrent or Metastatic Gastric or Gastroesophageal Junction Adenocarcinoma (KEYNOTE-059)	Merck Sharp & Dohme Corp.	PD-1	Pembrolizumab	2	Recruiting	Study first received: February, 2015 Last updated: July 16, 2015

NCT02340975	A Phase 1b/2 Study of MEDI4736 with Tremelimumab, MEDI4736 or Tremelimumab Monotherapy in Gastric or GEJ Adenocarcinoma	MedImmune LLC	PD-L1 CTLA-4	MEDI4736 (Anti-PD-L1) Tremelimumab (Anti CTLA-4)	1 & 2	Recruiting	Study first received: January 14, 2015 Last updated: May 15, 2015

NCT01975831	A Phase 1 Study to Evaluate the Safety and Tolerability of Anti-PD-L1, MEDI4736, in Combination with Tremelimumab in Subjects with Advanced Solid Tumors	Ludwig Institute for Cancer Research	PD-L1 CTLA-4	MEDI4736 (Anti-PD-L1) Tremelimumab (Anti CTLA-4)	1	Not yet recruiting	Study first received: December, 2013 Estimated completion date: December, 2016

NCT01585987	A Randomized, Open-Label, Two-Arm Phase II Trial Comparing the Efficacy of Sequential Ipilimumab versus Best Supportive Care Following First-Line Chemotherapy in Subjects with Unresectable Locally Advanced/Metastatic Gastric or Gastro-Esophageal Junction Cancer	Bristol-Myers Squibb	CTLA-4	Ipilimumab	2	Completed	Study first received: April 25, 2012 Completed: April, 2015
